# MultiParametric Magnetic Resonance Imaging-Based Nomogram for Predicting Prostate Cancer and Clinically Significant Prostate Cancer in Men Undergoing Repeat Prostate Biopsy

**DOI:** 10.1155/2018/6368309

**Published:** 2018-09-12

**Authors:** Cong Huang, Gang Song, He Wang, Guangjie Ji, Jie Li, Yuke Chen, Yu Fan, Dong Fang, Gengyan Xiong, Zhongcheng Xin, Liqun Zhou

**Affiliations:** ^1^Department of Urology, Peking University First Hospital, Beijing 100034, China; ^2^Institute of Urology, Peking University, National Urological Cancer Center of China, Beijing 100034, China; ^3^Department of Radiology, Peking University First Hospital, Beijing 100034, China; ^4^Department of Urology, Lishui Central Hospital, The Fifth Affiliated Hospital, Wenzhou Medical University, Lishui, 323000, Zhejiang, China; ^5^Department of Andrology, Peking University First Hospital, Beijing 100034, China

## Abstract

**Objective:**

To develop and internally validate nomograms based on multiparametric magnetic resonance imaging (mpMRI) to predict prostate cancer (PCa) and clinically significant prostate cancer (csPCa) in patients with a previous negative prostate biopsy.

**Materials and Methods:**

The clinicopathological parameters of 231 patients who underwent a repeat systematic prostate biopsy and mpMRI were reviewed. Based on Prostate Imaging and Reporting Data System, the mpMRI results were assigned into three groups: Groups “negative,” “suspicious,” and “positive.” Two clinical nomograms for predicting the probabilities of PCa and csPCa were constructed. The performances of nomograms were assessed using area under the receiver operating characteristic curves (AUCs), calibrations, and decision curve analysis.

**Results:**

The median PSA was 15.03 ng/ml and abnormal DRE was presented in 14.3% of patients in the entire cohort. PCa was detected in 75 patients (32.5%), and 59 (25.5%) were diagnosed with csPCa. In multivariate analysis, age, prostate-specific antigen (PSA), prostate volume (PV), digital rectal examination (DRE), and mpMRI finding were significantly independent predictors for PCa and csPCa (all* p* < 0.01). Of those patients diagnosed with PCa or csPCa, 20/75 (26.7%) and 18/59 (30.5%) had abnormal DRE finding, respectively. Two mpMRI-based nomograms with super predictive accuracy were constructed (AUCs = 0.878 and 0.927,* p* < 0.001), and both exhibited excellent calibration. Decision curve analysis also demonstrated a high net benefit across a wide range of probability thresholds.

**Conclusion:**

mpMRI combined with age, PSA, PV, and DRE can help predict the probability of PCa and csPCa in patients who underwent a repeat systematic prostate biopsy after a previous negative biopsy. The two nomograms may aid the decision-making process in men with prior benign histology before the performance of repeat prostate biopsy.

## 1. Introduction

Unlike other genitourinary tumours, prostate cancer (PCa) is often multifocal and heterogeneous and thus presents a challenge with regard to identifying malignant regions [1]. Prostate biopsy still remains the standard method for the diagnosis of PCa [2]. Despite advances in biopsy technology in recent decades, in approximately 60–70% of patients initial systematic prostate biopsy results are negative, due to the very limited or randomized sampling associated with the method [3]. In many of these patients a clinical suspicion of PCa persists, however, based on sustained elevated serum prostate-specific antigen (PSA), abnormal digital rectal examination (DRE) findings and/or suspicious lesions on magnetic resonance imaging (MRI). This poses a significant clinical dilemma for urologists, who must endeavour to identify the patients with negative pathology results who may be more likely to have PCa, in order to schedule them for a repeat prostate biopsy.

Due to the limitations of PSA associated with its noncancer specificity [4], the development of predictive models such as nomograms is of great interest. To date, several nomograms have been developed based on traditional clinicopathological parameters such as serum PSA, free/total PSA ratio (f/t), DRE, and prostate volume (PV) [5–7]. However, those traditional risk predictors do not correlate well with tumour aggressiveness [8]. And the utilization of these models for predicting PCa of subsequent biopsy has diminished over time,* as* screening has become more widespread in the general population.

Recently, the advances in multiparametric MRI (mpMRI) and the establishment of Prostate Imaging and Reporting and Data System (PI-RADS) criteria version 2 [9, 10] have facilitated visual identification and characterization of PCa, resulting in substantial improvements in diagnostic accuracy. Besides, several studies have showed that clinical nomograms incorporating mpMRI information improved early detection of PCa [11, 12]. However, none to date have specially aimed at patients with a previous negative prostate biopsy.

Thus, the study aimed to develop and internally validate two mpMRI-based nomograms for predicting the probability of PCa and clinically significant prostate cancer (csPCa) in patients undergoing repeat prostate biopsy. The two predictive nomograms were developed and may aid urologists when choosing patients with a previous negative prostate biopsy to undergo repeat biopsy.

## 2. Materials and Methods

### 2.1. Patient Cohort

After obtaining appropriate Institutional Review Board approval, we retrospectively reviewed the clinical data of 328 patients who underwent repeat transrectal ultrasound- (TRUS-) guided prostate biopsy after initial negative biopsy between January 2007 and April 2017 at our institution. The exclusion criterion was as follows: (1) previous diagnosis of PCa (n = 3); (2) medicated with 5-alpha reductase inhibitors (n = 13); (3) prior transurethral resection of the prostate (n = 7); (4) interval between prostate biopsies was ≤ 3 months (n = 57); and (5) incomplete data (n = 17). Finally, a total of 231 patients were enrolled in the study.

### 2.2. Clinicopathological Characteristics

All patient data were collated, including clinical and pathological characteristics. Age and body mass index at biopsy were calculated for each patient. Total serum PSA level and f/t ratio were measured before DRE and TRUS-guided biopsy. DRE findings were categorised as normal or abnormal. PV was measured by TRUS before performing the prostate biopsy and was calculated using the ellipsoid formula (width × length × height × 0.52). PSA density (PSAD) was calculated by dividing total PSA by PV on repeat biopsy. PSA velocity (PSAV) was calculated by dividing the time between the first and last biopsy by the change in PSA. A suspicious TRUS result was defined as any hypoechoic nodules in the peripheral zone [13]. Family history of PCa was not considered in the final analysis, due to an extremely low prevalence.

### 2.3. Biopsy Procedure and Histopathology

Most patients were counselled to undergo repeat biopsy more than 3 months after their initial biopsy, but some underwent a second biopsy ≤ 3 months thereafter due to anxiety or other reasons. These patients had already been excluded from this study based on the exclusion criteria. All patients underwent TRUS-guided systematic biopsies (114 cases of 6 district classic12-pin scheme, and 117 cases of 5 district classic 13-pin scheme), with the addition of two targeted biopsies at any area suspected of malignancy by ultrasonography. All biopsy specimens were evaluated by two dedicated genitourinary pathologists, in order to determine the cancer diagnosis and the Gleason score in positive cases. csPCa was defined as a Gleason score ≥ 7 (including Gleason scores 3 + 4, 4 + 3, 8, 9, and 10).

### 2.4. mpMRI

MRI was performed using a 1.5T or 3.0T whole-body system (GE Healthcare, USA) and a pelvic array coil (no endorectal coil was used). The imaging protocol included axial T1-weighted images of the pelvis and biplanar T2-weighted fast spin-echo images centred on the prostate. In addition, axial diffusion weighted imaging was performed with b-values of 0, 800, and 1000 sec/mm^2^. Dynamic contrast-enhanced images were performed following intravenous administration of gadolinium-chelate. The MRI acquisition details are summarized in Supplementary Table 1.

### 2.5. MRI Interpretation

MRI images were retrospectively interpreted by one of two experienced radiologists with > 5 years' experience reading prostate MRIs. Any disagreement in the process of interpretation was resolved by the adjudicating senior radiologist. The probability of tumour was evaluated and scored on a three-point scale based on PI-RADS version 2 scoring, where group “negative” (PI-RADS 1–2) = low probability, group “suspicious” (PI-RADS 3) = equivocal, and group “positive” (PI-RADS 4–5) = high or very high probability [9, 10].

### 2.6. Statistical Analysis

The primary endpoints of the study were identification of the presence of PCa and csPCa on repeat biopsy. Univariate analyses were performed to investigate associations between clinical and pathological risk factors and the presence of PCa or csPCa. Continuous variables were compared using Student's* t*-test and the Mann–Whitney U test, and categorical variables were compared using Pearson's test and Chi square test, as appropriate. Multivariate binary logistic regression analysis was conducted to identify independent predictors of the detection of PCa or csPCa.

We constructed two models for the prediction of the diagnosis of PCa and csPCa, using selected variables based on the results of multivariate binary logistic regression analysis. Discrimination was measured using the area under the curve (AUC) derived from the receiver operating characteristic (ROC) curves. Calibration curves to assess the performances of the two models were generated via bootstrap analysis with 150 resamples, to assess predicted vs. actual probability of PCa and csPCa. Decision curve analysis (DCA) as described by Vickers et al. was performed to evaluate the clinical utility of the two models by calculating the net benefits at a spectrum of probability thresholds [14].

Statistical analysis was performed using SPSS version 20.0 (IBM Corporation, Armonk, NY) and R version 3.1.3 (R foundation for Statistical Computing, Vienna, Austria). All analyses were two-sided, with statistical significance set at* p *< 0.05.

## 3. Results and Discussion

### 3.1. Baseline Clinicopathological Characteristics of the Study Cohort

The baseline clinical and pathological features of the entire cohort are summarized in Table 1. The median age was 70 years, and the interquartile range (IQR) was 64–74 years. The median prebiopsy PSA value was 15.03 ng/ml (IQR 10.03–23.15 ng/ml). All 231 men had repeat prostate biopsy after initial diagnosis of benign diseases, with a median time to repeat biopsy of 20.1 months (IQR 7.9–37.0 months). Abnormal DRE finding was presented in 14.3% of patients (n = 33) in the entire cohort. On repeat biopsy, PCa was detected in 75 patients (32.5%), and 59 (25.5%) were diagnosed with csPCa.

### 3.2. Univariate and Multivariate Analyses

In the univariate analysis, men diagnosed with PCa had statistically significant older age (72.87 vs. 67.19,* p* < 0.001), elevated PSA (22.32 vs. 13.44,* p* < 0.001), lower f/t (0.12 vs. 0.14,* p* = 0.016), lower PV (49.36 vs. 79.35,* p* < 0.001), higher PSAD (0.51 vs. 0.19,* p* < 0.001), higher PSAV (4.04 vs. 1.06,* p* < 0.001), more often abnormal DRE (26.7% vs. 8.3%,* p *< 0.001), more often suspicious TRUS (32.0% vs. 17.9%,* p* < 0.017), and more often suspicious/positive mpMRI finding (69.3% vs. 19.9%,* p* < 0.001) compared to men with negative biopsy. Similarly, older age (73.42 vs. 67.53,* p* < 0.001), elevated PSA (24.43 vs. 13.44,* p* < 0.001), lower f/t (0.12 vs. 0.14,* p* = 0.007), lower PV (52.48 vs. 80.92,* p* < 0.001), higher PSAD (0.58 vs. 0.20,* p* < 0.001), higher PSAV (5.29 vs. 1.11,* p* < 0.001), more often abnormal DRE (30.5% vs. 8.7%,* p *< 0.001), more often suspicious TRUS (35.6% vs. 18.0%,* p* < 0.005), and more often suspicious/positive mpMRI finding (79.6% vs. 21.0%,* p* < 0.001) were significantly associated with the presence of csPCa. Of note, those who were diagnosed with PCa or csPCa on repeat biopsy, 20/75 (26.7%) and 18/59 (30.5%) patients, had abnormal DRE finding (Table 1).

Besides, the association between mpMRI and tumour characteristics was showed in Table 2. Among patients with positive repeat prostate biopsy (n = 75), a higher PI-RADS was significantly associated with increasing biopsy Gleason grade group (*p* = 0.020) and clinical T–stage (*p* < 0.001) and both (*p* < 0.001). While among patients who underwent repeat prostate biopsy and radical prostatectomy (n = 30), mpMRI results using PI-RADS scheme were significantly correlated with increasing pathological Gleason grade group (*p* = 0.027), but not with pathological T–stage (*p* = 0.877).

In the multivariate analysis, age, PSA, PV, DRE, and mpMRI grade remained statistically significantly associated (all* p *< 0.01), suggesting that these variables were independent risk predictors for the diagnosis of overall PCa and csPCa on repeat biopsy (Table 3).

### 3.3. Development of Nomograms and Calibration Curve Analyses

**Figure 1:**
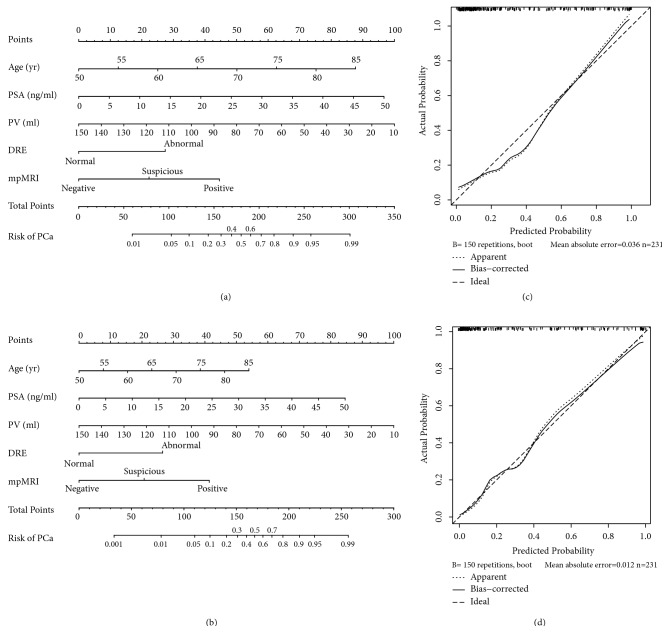
Nomogram (a) and calibration plot (c) to predict the probability of prostate cancer, and nomogram (b) and calibration (d) for prediction of clinically significant prostate cancer in patients who underwent repeat prostate biopsy.* Instructions.* Locate the patient variable value at each axis. Draw a vertical line up to the points axis to identify how many points are attributed for each variable value. Sum the points for all variables. Locate the final sum on the total points axis, and draw a straight line down to assess the individual probability of PCa or csPCa in repeat prostate biopsy.

Based on the above multivariate analysis results, two predictive nomograms for overall PCa and csPCa were constructed using selected risk factors including age, PSA, PV, DRE, and mpMRI results (Figures 1(a) and 1(b)). The two nomograms were internally validated in our cohort. Two calibration plots were constructed using a database of 150 resamples, in order to measure the fit between the predicted rate and the actual outcome. A perfect model would demonstrate a 1:1 relationship between predicted and observed values, resulting in a perfect 45° slope. Our models for predicting the presence of PCa and csPCa exhibited excellent calibration (Figures 1(c) and 1(d)).

### 3.4. ROC Analysis and DCA

**Figure 2:**
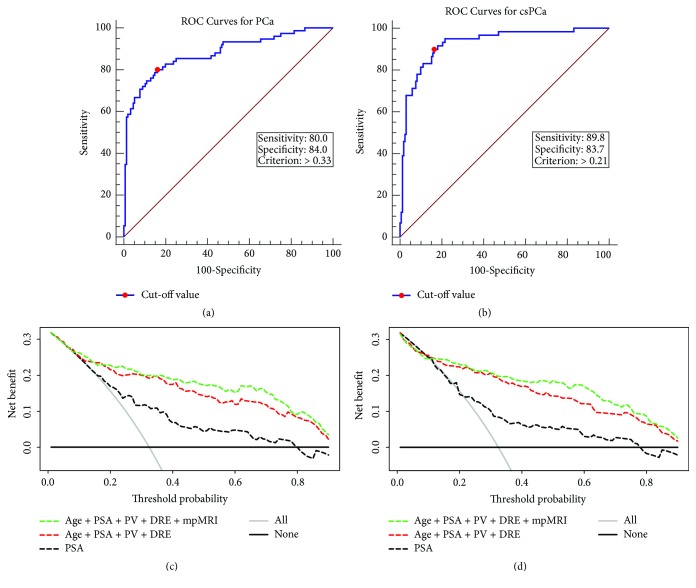
Receiver operating characteristic (ROC) curves of the nomograms for predicting PCa (a) and csPCa (b), with the AUC value of 0.878 and 0.927. Decision curve analysis (DCA) of the nomograms for prediction of PCa (c) and csPCa (d), which demonstrated a high net benefit across a wide range of threshold probabilities.

ROC analysis was performed to assess the accuracy of the two models. The nomogram applied for overall PCa yielded an AUC of 0.878, with a 95% confidence interval (CI) of 0.8290.917 (*p* < 0.001). The model for the evaluation of csPCa yielded an AUC of 0.927, with a 95% CI of 0.886–0.957 (*p* < 0.001) (Figures 2(a) and 2(b)). According to the two ROC curves, the most discriminative probability cutoff points of two nomograms were 0.33 and 0.21, with taking into consideration an appropriate trade-off between the sensitivity and specificity.

**Figure 3:**
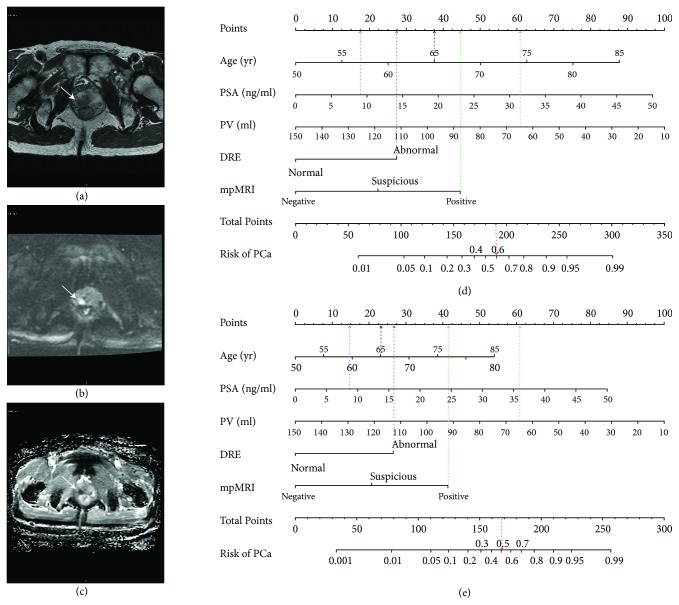
A 65-year-old patient with an elevated PSA of 8.58 ng/ml and a previous negative biopsy, which was diagnosed with a Gleason 4 + 3 = 7 prostate cancer on repeat biopsy.** (a)** Axial T2-weighted axial image showing a lesion of low T2 signal in the right mid peripheral zone.** (b)** Diffusion weighted imaging with b value of 1000 sec/mm2 confirming the lesion.** (c)** Apparent diffusion coefficient map shows a focal area of diffusion restriction, measuring 1.3 cm in the longest diameter (white arrow). The total PI-RADS version 2 score for the lesion was 4 according to both radiologists, which is suggestive of a high probability of prostate cancer.** (d)** Nomogram predicting probability of PCa for this patient. The corresponding points for the variables (age, 65 years = 38 points [black line]; PSA, 8.58 ng/ml = 18 points [pink line]; PV, 64.25 ml = 61 points [grey line]; DRE, abnormal = 28 points [blue line]; mpMRI, positive = 45 points [green line]) yielding a total of 190 points, which indicates the probability of having PCa is 0.60 [red line]. For a probability > 0.33 defined as being compatible with PCa, nomogram correctly predicted the presence of PCa.** (e)** Nomogram predicting probability of csPCa for this patient. Similarly, a total of 168 points showed that the probability of detecting csPCa is 0.50, greater than the cutoff of 0.21, which allowed correct prediction of the presence of csPCa.

DCAs for evaluating the clinical utility of the two predictive models were plotted (Figures 2(c) and 2(d)). The mpMRI-based nomograms showed the highest net benefits across the entire spectrum of probability thresholds, indicating that our nomograms were superior to using PSA alone or a PSA-based risk model. The examples of the diagnostic use of the two nomograms were shown in Figure 3.

### 3.5. Discussion

Systematic TRUS-guided prostate biopsy remains the recommended investigation for the histological diagnosis of PCa [2]. However, in approximately 60-70% of patients who are thought to harbour malignancy, PCa cannot be definitively diagnosed based on their first prostate biopsy [3]. Following negative biopsy, many of these patients continue to exhibit a clinical constellation consistent with PCa based on persistently elevated serum PSA, abnormal DRE findings, and/or suspicious mpMRI. Thus, identifying appropriate candidates for repeat biopsy in such situations poses a clinical dilemma for urologists.

It is well known that the detection rate of subsequent prostate biopsies is lower than that of initial biopsies [15–17]. In a prospective study of over 2500 men undergoing repeat biopsies, the serial positive rates were 29%, 17%, 14%, 11%, 9%, and 7% on each subsequent biopsy [18]. In the cohort in the current study, the detection rates of PCa and csPCa were 32.5% and 25.5%, a much higher proportion than most previous studies. Additionally, the median serum PSA value of the patients in the current study was 15.03 ng/ml, higher than all previously reported cohorts. Of those patients with PCa or csPCa, 20/75 (26.7%) and 18/59 (30.5%) had abnormal DRE finding. This may be due to the lack of a PSA-based PCa screening programme in the Chinese population studied.

Over the past few years, several models have been constructed in efforts to predict cancer risk among men with previous negative biopsies, including nomograms described by Yanke et al. [19] and Moussa et al. [20]. These models were mostly developed on the basis of screening of European or American populations, and integrating various PSA-based clinicopathological variables yielded better results than any single risk factor alone. Nonetheless, very few studies have investigated the application of mpMRI in multivariate nomograms to predict PCa and csPCa in repeat prostate biopsies. Thus, there is a need for new predictive models incorporating mpMRI information.

Most current practice guidelines do not recommend mpMRI for men before the first prostate biopsy, whereas they do acknowledge that mpMRI examination may increase the detection of clinically significant cancer [2, 21, 22]. The performance of prebiopsy mpMRI is recognized, but it is not widely used in Europe or the United States as it is not covered by medical insurance. In the study, all patients underwent mpMRI prior to repeat biopsy. Older age, higher PSA, smaller PV, abnormal DRE, and positive mpMRI were independent risk factors for the detection of overall PCa and csPCa. On that basis, we constructed two nomograms based on the large cohort from a single institution, in order to predict the probabilities of malignancy diagnosis on repeat biopsy. The two nomograms performed favourably compared with most previous reports, with AUCs of 0.878 and 0.927 [19, 20, 23]. Such high AUCs may be partially due to the incorporation of mpMRI information into the models in the current study.

In a recently published critical meta-analysis involving nearly 2000 patients from twelve different studies, researchers assessed the diagnostic accuracy of mpMRI for the detection of clinically significant PCa [24]. They reported that cancer detection rates ranged from 44% to 87%, with negative predictive values ranging from 63% to 98%. Pepe et al. [25] analysed 100 patients who underwent repeat biopsy for persistently elevated PSA and reported that mpMRI was significantly correlated with aggressiveness of PCa. Mendhiratta et al. [26] concluded that sparing biopsies in men with PI-RADS scores of < 4 may warrant consideration, due to the relatively low rate of detecting Gleason score ≥ 7 cancer. In addition, Pepe et al. [27] also showed that the detection rate of csPCa was 72.7%, 87%, and 100% for patients with a PI-RADS score of 3, 4, and 5. The presence of a PI-RADS of 5 is suspicious of a csPCa in about all the cases. In the current study, the respective positive and negative predictive values of mpMRI for the diagnosis of overall PCa were 69.3% and 79.6%, and for csPCa they were 80.1% and 79.1%. These results are concordant with previous reports that the use of mpMRI could improve the ability to detect PCa, particularly csPCa, in men with a negative diagnosis on analysis of biopsy samples.

The predictive value of PSAD and PSAV for cancer detection on repeat prostate biopsy evidently remains controversial in the literature. Recently, a meta-analysis reported limited predictive benefits using PSA kinetics, known as PSA velocity and PSA doubling time, and did not recommend the use of these PSA kinetics to determine the need for repeat biopsy [28]. Similarly, PSAD and other such measures have not been demonstrated to be of predictive value [29]. The TRUS technique is recommended as the method of choice for biopsy guidance in general, whereas its poor sensitivity in diagnosing malignancy due to the invisibility of some lesions has consistently proved to be a weakness, as has high interobserver variability [30]. Several studies suggest that typical TRUS-guided biopsy methods have respective sensitivity and specificity profiles of 60–80% and 82–100%, with associated false negative rates of 20–40% [31–33]. In the current study, in multivariate analysis PSAD, PSAV, and TRUS imaging were not independently significant predictors of PCa or csPCa. Thus, we did not include those variables in our final models.

The current study had several limitations. First, it was a retrospective study with a probable risk of selection bias. Second, the predictive models constructed were based on a relatively small sample size from a single institution. Third, all patients underwent TRUS-guided systematic prostate biopsy during the time of reviewed, with no data regarding the diagnostic accuracy of MRI/TRUS fusion targeted biopsy. In addition, the patients had a relatively higher PSA value plus a comparatively higher positive rate of DRE finding, may select a population with a high risk for PCa, and reduce the clinically accuracy of mpMRI. Moreover, owing to their relative rarity the two models did not include novel molecular markers such as prostate cancer antigen 3 [34], and we did not utilize the Prostate Health Index [35]. Finally, our nomograms require external validation in a multicentre study to assess their wider applicability.

## 4. Conclusion

mpMRI combined with age, serum PSA, PV, and DRE can help to predict the probabilities of PCa and csPCa in patients who underwent a repeat systematic prostate biopsy after a previous negative biopsy. The two nomograms constructed in the current study may aid in decision-making for men with prior benign histology before the performance of repeat biopsy.

## Figures and Tables

**Table 1:** Baseline Clinical and Demographic characteristics of the study cohort.

Variable	Total	PCa			csPCa		
		NO	YES	*p *value	NO	YES	*p *value
Patients, No. (%)	231 (100)	156 (67.5)	75 (32.5)		172 (74.5)	59 (25.5)	
Interval, Median (IQR), m	20.1 (7.9-37.0)						
Age, Mean ± SD, years	69.03 ± 7.05	67.19 ± 6.60	72.87 ± 6.41	< 0.001	67.53 ± 6.68	73.42 ± 6.25	<0.001
BMI, Mean ± SD, kg/ml	23.93 ± 2.79	23.90 ± 2.69	24.00 ± 3.00	0.796	23.85 ± 2.71	24.16 ± 3.01	0.476
PSA, Median (IQR), ng/ml	15.03 (10.03-23.15)	13.44 (9.44-17.93)	22.32 (13.77-36.89)	< 0.001	13.44 (9.48-18.59)	24.43 (15.89-42.84)	<0.001
f/t, Mean ± SD, %	0.14 ± 0.06	0.14 ± 0.06	0.12 ± 0.07	0.016	0.14 ± 0.06	0.12 ± 0.07	0.007
PV, Mean ± SD, ml	71.69 ± 42.41	80.92 ± 46.04	52.48 ± 24.40	< 0.001	79.35 ± 45.25	49.36 ± 20.34	<0.001
PSAD, Median (IQR), ng/ml/ml	0.25 (0.15-0.45)	0.19 (0.12-0.29)	0.51 (0.25-0.76)	< 0.001	0.20 (0.12-0.30)	0.58 (0.33-0.79)	<0.001
PSAV, Median (IQR), ng/ml/yr	1.55 (0.12-5.43)	1.06 (-0.64-3.74)	4.04 (1.38-8.60)	< 0.001	1.11 (-0.24-4.04)	5.29 (1.52-9.09)	< 0.001
DRE, No. (%)				< 0.001			< 0.001
Normal	198 (85.7)	143 (91.7)	55 (73.3)		157 (91.3)	41 (69.5)	
Abnormal	33 (14.3)	13 (8.3)	20 (26.7)		15 (8.7)	18 (30.5)	
TRUS, No. (%)				0.017			0.005
Negative	179 (77.5)	128 (82.1)	51 (68.0)		141 (82.0)	38 (64.4)	
Positive	52 (22.5)	28 (17.9)	24 (32.0)		31 (18.0)	21 (35.6)	
mpMRI, No. (%)				< 0.001			< 0.001
Negative	148 (64.1)	125 (80.1)	23 (30.7)		136 (79.1)	12 (20.3)	
Suspicious	31 (13.4)	16 (10.3)	15 (20.0)		18 (10.5)	13 (22.0)	
Positive	52 (22.5)	15 (9.6)	37 (49.3)		18 (10.5)	34 (57.6)	

PCa, prostate cancer; csPCa, clinically significant prostate cancer; IQR, interquartile range; SD, standard deviation; BMI, body mass index; PSA, prostate-specific antigen; f/t, free/total PSA ratio; PV, prostate volume; PSAD, PSA density; PSAV, PSA velocity; DRE, digital rectal examination; TRUS, transrectal ultrasound; mpMRI, multi-parametric magnetic resonance imaging.

**Table 2:** Association between mpMRI and tumor characteristics in patients with positive repeat prostate biopsy (n = 75) or in patients with both repeat prostate biopsy and radical prostatectomy (n = 30).

	Gleason Grade Group, GGG					Clinical or Pathological T-stage			Total
	GGG 1	GGG 2	GGG 3	GGG 4	GGG 5	T1	T2	T3	
Patients with positive repeat prostate biopsy (n = 75)									

PI-RADS 1-2	11 (50.0%)	5 (22.7%)	0 (0.0%)	4 (18.2%)	2 (9.1%)	16 (72.7%)	6 (27.3%)	0 (0.0%)	22
PI-RADS 3	2 (13.3%)	6 (40.0%)	1 (6.7%)	3 (20.0%)	3 (20.0%)	0 (0.0%)	15 (100.0%)	0 (0.0%)	15
PI-RADS 4-5	3 (7.9%)	11 (28.9%)	6 (15.8%)	8 (21.1%)	10 (26.3%)	0 (0.0%)	28 (73.7%)	10 (26.3%)	38
Total	16 (21.3%)	22 (29.3%)	7 (9.3%)	15 (20.0%)	15 (20.0%)	16 (21.3%)	49 (65.3%)	10 (13.3%)	75

Patients with both repeat prostate biopsy and radical prostatectomy (n = 30)									

PI-RADS 1-2	4 (30.8%)	2 (15.4%)	4 (30.8%)	1 (7.7%)	2 (15.4%)	-	8 (61.5%)	5 (38.5%)	13
PI-RADS 3	0 (0.0%)	4 (80.0%)	0 (0%)	0 (0.0%)	1 (20.0%)	-	3 (60.0%)	2 (40.0%)	5
PI-RADS 4-5	0 (0.0%)	1 (8.3%)	3 (25.0%)	4 (33.3%)	4 (33.3%)	-	6 (50.0%)	6 (50.0%)	12
Total	3 (10.0%)	8 (26.7%)	7 (23.3%)	5 (16.7%)	7 (23.3%)	-	17 (56.7%)	13 (43.3%)	30

PI-RADS: Prostate Imaging Reporting and Data System.

**Table 3:** Multivariate analysis of predictors associated with PCa and csPCa.

Variable	PCa				csPCa			
	OR	95% CI	*p *value	AUC	OR	95% CI	*p *value	AUC
Nomograms		0.83-0.92	< 0.001	0.878		0.87-0.96	< 0.001	0.927
Age, yr	1.09	1.03-1.16	0.005		1.08	1.00-1.16	0.028	
PSA, ng/ml	1.06	1.03-1.10	0.001		1.07	1.03-1.12	0.001	
f/t, %			0.828				0.574	
PV, ml	0.97	0.96-0.98	< 0.001		0.97	0.95-0.99	< 0.001	
PSAD, ng/ml/ml			0.138				0.421	
PSAV, ng/ml/yr			0.525				0.406	
DRE	3.2	1.08-9.41	0.035		4.37	1.39-13.80	0.012	
TRUS			0.200				0.245	
mpMRI								
Negative	1				1			
Suspicious	3.27	1.12-9.52	0.030		4.78	1.47-15.52	0.009	
Positive	5.82	2.34-14.46	< 0.001		8.41	3.14-22.50	< 0.001	

PCa, prostate cancer; csPCa, clinically significant prostate cancer; OR, Odds Ratio; CI, confidence interval; AUC, area under the receiver operating characteristics curve; PSA, prostate-specific antigen; f/t, free/total PSA ratio; PV, prostate volume; PSAD, PSA density; PSAV, PSA velocity; DRE, digital rectal examination; TRUS, transrectal ultrasound; mpMRI, multi-parametric magnetic resonance imaging.

## Data Availability

The data used to support the findings of this study are available from the corresponding author upon request.

## Supplementary Materials

Supplementary Materials Supplementary Table 1: The prostate MRI parameters at 1.5T or 3.0T.
