# Supplementary material for: MultiParametric Magnetic Resonance Imaging-Based Nomogram for Predicting Prostate Cancer and Clinically Significant Prostate Cancer in Men Undergoing Repeat Prostate Biopsy
Source: Biomed Res Int. 2018 Sep 12;2018:6368309. doi: 10.1155/2018/6368309 (PMC6157114; doi:10.1155/2018/6368309)
Supplement: Supplementary Materials — Supplementary Table 1: The prostate MRI parameters at 1.5T or 3.0T. [file 6368309.f1.docx]

**Supplementary Table 1.** The prostate MRI parameters at 1.5T or 3.0T.

|  | The prostate MRI parameters | | | | | | |
| --- | --- | --- | --- | --- | --- | --- | --- |
|  | 1.5T | | |  | 3.0T | | |
| Parameters | T2WI | DWI | DCE |  | T2WI | DWI | DCE |
| Repetition time (msec) | 3500 | 3500 | 4 |  | 2900 | 4000 | 3.3 |
| Echo time (msec） | 85 | 56.4 | 1.9 |  | 90 | 70 | 1.6 |
| Flip angle (degree) | 90, 180 | 90 | 15 |  | 90, 180 | 90 | 15 |
| Matrix | 320×256 | 128×128 | 256×256 |  | 320×280 | 184×184 | 256×256 |
| Field of view (cm^2^) | 24 | 26 | 36 |  | 26 | 26 | 26 |
| No. of temporal acquisitions |  |  |  |  | 4 | 4 | 0.75 |
| Section thickness (mm) | 4 | 5 | 3.8 |  | 4 | 4 | 2 |
| b value (sec/mm^2^) | - | 0, 800 | - |  | - | 0, 800, 1000 | - |
